# Potential Innovative Tools for Heritage Conservation: A Novel RNA-FISH Probe and Antimicrobial Peptides for the Detection and Control of *Arthrobacter* spp.

**DOI:** 10.3390/microorganisms14030687

**Published:** 2026-03-18

**Authors:** Patrícia Branco, Ana Teresa Caldeira, Marina González-Pérez

**Affiliations:** 1 BIORG—Bioengineering and Sustainability Research Group, Lusófona University, Av. Campo Grande 376, 1749-024 Lisboa, Portugal; 2 HERCULES Laboratory—Cultural Heritage, Studies and Safeguarding & IN2PAST—Associate Laboratory for Research and Innovation in Heritage, Arts, Sustainability and Territory, University of Évora, Largo Marquês de Marialva 8, 7000-809 Évora, Portugal; atc@uevora.pt (A.T.C.);; 3 Linking Landscape, Environment, Agriculture and Food (LEAF), Associated Laboratory TERRA, Instituto Superior de Agronomia, University of Lisbon, Tapada da Ajuda, 1349-017 Lisbon, Portugal; 4 Chemistry and Biochemistry Department, School of Sciences and Technology, University of Évora, Rua Romão Ramalho 59, 7000-671 Évora, Portugal; 5 Institute of Urban and Sustainable Development, City University of Macau, Avenida Padre Tomás Pereira, Taipa, Macau

**Keywords:** biodeterioration, *Arthrobacter* spp., fluorescence *in situ* hybridisation, RNA-FISH probes, antimicrobial peptides, heritage conservation

## Abstract

Microorganisms such as *Arthrobacter* spp. are important agents of biodeterioration in cultural heritage (CH) environments, causing orange–yellow chromatic alterations and contributing to substrate degradation. This study evaluates two complementary tools for the rapid detection and mitigation of *Arthrobacter* spp.: a newly designed genus-specific RNA–fluorescence *in situ* hybridisation (FISH) probe (Art1420-Cy3) and an antimicrobial peptide fraction produced by *Saccharomyces cerevisiae* ISA 1028. The RNA-FISH probe Art1420-Cy3 showed high specificity and sensitivity, labelling 80–85% of *Arthrobacter* cells at 10% (*v*/*v*) formamide and enabling their detection by epifluorescence microscopy and flow cytometry. The peptide fraction exhibited pronounced bactericidal activity, reducing *Arthrobacter* culturability from ~10^8^ to ~10^1^ CFU/mL within 48 h, while also inhibiting other biodeteriogenic microorganisms. Overall, these findings outline the basis for an integrated and CH-compatible approach that combines precise *Arthrobacter* cells detection and identification with targeted, biologically derived control. Although further validation using real heritage samples and application protocols specifically tailored to sensitive materials is required, this strategy shows strong potential as a sustainable alternative to conventional chemical biocides and as a practical framework for detecting and mitigating pigment-producing biodeteriogens in CH and other vulnerable environments.

## 1. Introduction

Microorganisms, including bacteria, filamentous fungi, yeasts, and algae, play a crucial role in the biodeterioration of cultural heritage (CH) assets due to their metabolic diversity and adaptability to extreme environments. These microorganisms produce a wide range of metabolites that can affect heritage materials; among them, pigments are often the most visually evident, as they can cause noticeable chromatic alterations that compromise the aesthetic integrity of artworks [1,2,3,4]. The establishment of specific microbial communities on CH materials depends on multiple factors, including environmental conditions, the organic or inorganic composition of the substrate, and its surface characteristics [5].

Among biodeteriogenic microorganisms, filamentous fungi are the most frequently encountered, causing extensive aesthetic and structural damage to artworks such as wall paintings, sculptures, books, and manuscripts [1,6,7,8,9,10]. However, bacteria also play a significant role in CH deterioration, and among them, *Arthrobacter* spp. has gained increasing attention due to its ability to produce carotenoid pigments that induce orange–yellow chromatic alterations on artworks [4,11,12,13].

In this context, several studies have documented the presence of *Arthrobacter* species in diverse CH objects affected by biodeterioration, highlighting their ecological adaptability and widespread occurrence on various substrates [11,13,14,15]. *Arthrobacter* spp. were detected as the representative bacterium within the microbial community colonising oil painting, stone and mural paintings from historical sites [14,15,16].

Besides their role as biodeteriogenic agents, *Arthrobacter* spp. may also raise health and biosafety concerns. Some species have been reported to persist in indoor environments and have occasionally been isolated from clinical specimens, highlighting their potential relevance beyond cultural heritage deterioration [17,18,19,20].

In addition to bacteria, yeasts are also important contributors to the biodeterioration of CH materials. Pigmented yeasts of the genus *Rhodotorula* are frequently detected on stone, mortar, and painted surfaces, where their characteristic pink-to-orange hues result from the intracellular accumulation of carotenoid pigments such as torulene and torularhodin [21,22,23].

Considering that both *Arthrobacter* and *Rhodotorula* are pigmented microorganisms frequently detected on similar CH substrates, their colonisation clearly illustrates how microbial growth can severely compromise the aesthetic integrity of artworks [24]. Because both genera produce intracellular carotenoid pigments that give rise to comparable orange–pink chromatic alterations, the visual changes they induce are often indistinguishable, meaning that what appears to be yeast colonisation may, in fact, be bacterial. This overlap complicates diagnosis and can delay the selection and application of appropriate conservation treatments. Distinguishing between these pigment-producing taxa is therefore a critical challenge for conservation practice, as accurate identification underpins the choice of effective and compatible remediation strategies.

Although culture-based methods combined with optical microscopy can distinguish *Rhodotorula* from *Arthrobacter* under optimal laboratory conditions, their applicability in real cultural heritage (CH) scenarios is severely constrained by several well-documented factors. First, many bacteria, including environmental actinobacteria, frequently enter a viable but non-culturable (VBNC) state under oligotrophic and stress-prone conditions, leading to a substantial underestimation of viable cells when culture-dependent approaches are used [25]. Second, microbial communities on CH surfaces are predominantly organised as biofilms, whose extracellular polymeric matrices hinder microscopic penetration and compromise accurate cell enumeration [26,27]. Third, culture-based identification typically requires 48–72 h and selectively favours fast-growing microorganisms, whereas fluorescence *in situ* hybridisation (FISH) enables rapid detection and identification of target taxa within a few hours, including cells embedded in biofilms or present in VBNC states [28,29]. Finally, multiple comparative studies have demonstrated that culture-based methods consistently underestimate total microbial biomass and diversity, particularly in complex or stress-adapted communities, reinforcing the need for culture-independent molecular diagnostics for in situ analysis of heritage materials [30,31].

To overcome the limitations of conventional culture-based methods, faster, less labour-intensive, and more reliable analytical approaches have been increasingly adopted in recent years to characterise microbial communities involved in cultural heritage deterioration. These include Polymerase Chain Reaction (PCR)-based assays, genomic techniques, and FISH, which offer higher specificity, sensitivity, and accuracy than traditional culture-dependent methods [1,32,33,34,35,36,37]. PCR and genomic analyses have already enabled the detection of carotenoid-producing microorganisms in CH samples, including *Arthrobacter* spp. [16]. Among these molecular tools, the FISH technique provides a distinct advantage, as it allows *in situ* identification of biodeteriogenic microorganisms directly within the material, enabling visualisation of target cells in their native microenvironment [38,39,40]. Furthermore, FISH facilitates the molecular identification of individual microbial cells, overcoming the constraints of both culture-dependent and PCR-based methods. This technique relies on fluorescently labelled oligonucleotide probes that hybridise to specific DNA or RNA sequences of the target organism, resulting in fluorescent signals that permit direct single-cell detection and analysis via epifluorescence microscopy (EM) or flow cytometry (FC) [37,41,42,43,44,45]. In particular, RNA-FISH primarily detects viable or VBNC microbial cells, as RNA signals are typically strong in active cells but weak or absent in dead ones due to rapid RNA degradation *post-mortem*; this method can be combined with live/dead stains to confirm cell viability, supporting the interpretation of fluorescent signals as indicative of live cells [38]. Despite its proven applicability in identifying a wide variety of biodeteriogenic microorganisms, to our knowledge, no FISH probe has yet been developed for the detection of *Arthrobacter* spp.

At the same time, the control of biodeteriogenic microorganisms in CH settings remains challenging. Conventional mechanical treatments often fail to ensure complete eradication,. physical treatments may damage fragile materials and and most chemical biocides, whereas effective pose environmental and health hazards [46,47,48].

There is, therefore, a pressing need for biocompatible and sustainable biocidal alternatives that, being compatible with the preservation of the historic materials, allow to eliminate, inhibit and prevent microbiological contamination of cultural artefacts.

The search for safer and more sustainable antimicrobial approaches has led to increasing interest in bioactive compounds of microbial origin. Among these, *Saccharomyces cerevisiae* has been recognised as a source of low molecular weight antimicrobial peptides (AMPs) with activity against both bacterial and yeast species [49,50,51,52]. These AMPs are derived from cellular metabolism, particularly from the glycolytic enzyme glyceraldehyde-3-phosphate dehydrogenase (GAPDH). They have been shown to inhibit several microorganisms and therefore represent promising naturally derived alternatives to conventional antimicrobial agents [49]. Peptide fractions in the 2–10 kDa range, recovered from *S. cerevisiae* culture supernatants, have consistently shown antimicrobial activity against different microbial species but, in our knwoledge, none study has been previous assessed their antimicrobial potential against *Arthrobacter* spp.

Considering all of the above, the present study therefore aims to develop potential complementary tools for the detection and control of *Arthrobacter* spp. in CH environments. Specifically, we (i) designed and evaluated the performance of a novel genus-specific RNA-FISH probe (Art1420-Cy3) for the detection of *Arthrobacter* spp. and (ii) assessed the antimicrobial potential of a 2–10 kDa peptide fraction produced by *S. cerevisiae* ISA 1028 against these bacteria.

The design of an effective FISH genus-specific probe represents an important advancement, enabling precise identification of *Arthorbacter* spp. within complex microbial communities, thereby improving the accuracy of conservation diagnostics and supporting targeted intervention strategies [53].

Likewise, the exploration of sustainable alternatives to conventional chemical biocides is essential for mitigating biodeteriogenic microorganisms in cultural heritage contexts. In this regard, the evaluation of the 2–10 kDa peptide fraction produced by *S. cerevisiae* ISA 1028 may provide a promising biocontrol strategy for limiting the proliferation of *Arthrobacter* spp. and reducing their impact on heritage materials.

Together, these approaches provide novel complementary solutions for molecular detection and biocontrol of *Arthrobacter* spp. in CH environments. Additionally, the tools proposed here have potential applications in other sectors where *Arthrobacter* spp. contamination can affect material performance or safety, including industrial processes, bioremediation, and biomedical settings.

## 2. Materials and Methods

### 2.1. Strains and Growth Conditions

In this study, four bacterial strains isolated from CH assets [16] were used: *Arthrobacter* sp. CCLBH-BP301, *Arthrobacter* sp. CCLBH-BP302, and *Bacillus* sp. CCLBH-BP102 from the Culture Collection Laboratory of Biodegradation (HERCULES, University of Évora, Évora, Portugal), and *Rubrobacter radiotolerans* DSM 5868 (*R. radiotolerans*) from the Deutsche Sammlung von Mikroorganismen und Zellkulturen (DSMZ, Braunschweig, Germany).

In addition, three yeast strains, i.e., *Rhodotorula* sp. CCLBH-YMP502 and *Cryptococcus adeliensis* CCLBH-YMP503, also isolated from CH materials, and *Saccharomyces cerevisiae* ISA 1028, isolated from white wine from the culture collection of Instituto Superior de Agronomia (University of Lisbon, Lisboa, Portugal), were used.

All bacterial strains except *R. radiotolerans* were incubated on Nutrient Agar slants (3.0 g/L meat extract, 5.0 g/L peptone, and 15.0 g/L agar) at 30.0 °C for 48–72 h and then they were directly used or stored at 4.0 °C. The inocula were prepared by transferring biomass from the respective agar slants into 100 mL Erlenmeyer flasks containing 50 mL of liquid medium. Nutrient broth (NB) was the liquid medium used (1.0 g/L glucose, 15.0 g/L peptone, 6.0 g/L sodium chloride, and 3.0 g/L yeast extract) and the cultures were incubated at 30 °C with agitation at 150 rpm for 6 h to reach the exponential growth phase.

*R. radiotolerans* DSM 5868 were maintained on Tryptic Soy (TS) agar slants (15.0 g/L casein peptone (pancreatic), 5.0 g/L soya peptone (papainic), 5.0 g/L sodium chloride and 15.0 g/L agar) at 37.0 °C for 48–72 h and then they were directly used or stored at 4.0 °C. The inocula were prepared in TS medium containing 15.0 g/L casein peptone (pancreatic), 5.0 g/L soya peptone (papainic), and 5.0 g/L sodium chloride. The cultures were incubated at 37 °C under static conditions for 8 h to reach the exponential growth phase.

Yeasts were maintained in Yeast Peptone Dextrose (YEPD) agar slants (20 g/L bacteriological peptone, 20 g/L glucose, 10 g/L yeast extract, 15 g/L agar). They were incubated at 30.0 °C for 48–72 h, and then they were directly used or stored at 4.0 °C. Yeast growth was carried out by harvesting the cells from one fresh YEPD-agar slant and transferring them to 100 mL Erlenmeyer flasks containing 50 mL of YEPD liquid medium (20 g/L bacteriological peptone, 20 g/L glucose, 10 g/L yeast extract). Liquid cultures were incubated for 16 h at 28.0 °C and 120 rpm in an orbital shaker.

All media were sterilised by autoclaving at 120 °C for 20 min.

### 2.2. Design and In Silico Evaluation of Genus-Specific Probe for Arthrobacter spp.

To design a genus-specific RNA-FISH probe for *Arthrobacter* spp., ribosomal RNA (rRNA) sequences (16S rRNA) representing the target group were retrieved from the National Centre for Biotechnology Information (NCBI) database (https://www.ncbi.nlm.nih.gov/; [54]; accessed on 22 February 2024). The sequences were aligned using the BioEdit software package 7.2 (https://bioedit.software.informer.com; [55]) and subsequently analysed with the Design Probes web tool of the DECIPHER programme (https://decipher.codes/DesignProbes.html; [56]; accessed on 29 February 2024).

From the probes generated, ten candidates, those with the highest specificity scores and predicted hybridisation, were selected for further evaluation. The *in silico* assessment followed the methodology described by Branco et al. (2020) [45]. Briefly: (a) probe specificity was reconfirmed through BLASTN 2.16 searches on the NCBI platform; (b) single-stranded oligonucleotide properties, including molecular weight, melting temperature (Tm), GC content, inter-molecular self-complementarity, and potential intra-molecular hairpin formation, were calculated using OligoCalc, oligonucleotide properties calculator [57]; and (c) predicted FISH performance parameters namely hybridisation efficiency, probe affinity, formamide melting point ([FA]m), and FA dissociation profiles were estimated using mathFISH (http://mathfish.cee.wisc.edu; [58]).

Among all candidates, the probe displaying the highest predicted specificity and in silico FISH performance was selected for experimental validation.

### 2.3. Analyses of the Performance and Specificity of the Probes Designed

#### 2.3.1. RNA-FISH Probes

The RNA-FISH probes used in this work were labelled in the 5′ end. EUK516-Cy3 (an eukaryotic universal probe used as positive control for yeasts), EUB338-Cy3 (a universal eubacterial probe used as negative control for yeasts and positive control for bacteria), NONEUB338-Cy3 (control probe complementary to EUB338 used as negative control for bacteria), and the genus-specific probe for *Arthrobacter* spp., Art1420-Cy3 (L-S-Art1420-a-A-17), designed in this study.

#### 2.3.2. RNA-FISH Procedure

After incubation, cultured cells were collected by centrifugation (10,000 rpm, 10 min) and washed with Phosphate-Buffered Saline (PBS: 130.0 mM NaCl, 8.0 mM NaH_2_PO_4_, 2.7 mM KCl, 1.5 mM KH_2_PO_4_, pH 7.2). Cells were fixed in absolute ethanol for 1 h at room temperature to preserve cellular integrity while permeabilizing the membranes, washed again with PBS and stored in 50:50 EtOH/PBS (*v*/*v*) at −20.0 °C until use in FISH assays.

The subsequent steps of the in-suspension RNA-FISH protocol (hybridisation, washing and analysis) followed the procedure described by Branco et al. (2020) [45], with minor adaptations. Briefly, fixed cells (10^6^–10^8^ per assay) were resuspended in 80 µL hybridisation buffer (0.9 M NaCl, 20 mM Tris-HCl, 0.1% SDS, pH 7.2) containing the desired formamide concentration ([FA], 10%, *v*/*v*) and 1 µL of probe stock solution (120 ng/µL). Hybridisation was performed for 2 h at 46 °C in the dark with gentle agitation, followed by a 30 min wash in pre-warmed washing buffer with stringency adjusted to the [FA]% used in the hybridisation step. Finally, cells were pelleted, resuspended in 500 µL PBS and analysed by epifluorescence microscopy (EM) and flow cytometry (FC).

For all organisms and conditions, four types of RNA-FISH assays were run in parallel: (i) blanks (no probe; control for natural and FISH-induced autofluorescence); (ii) positive controls, using EUB338-Cy3 for bacteria or EUK516-Cy3 for yeasts; (iii) negative controls, using NONEUB338-Cy3 for bacteria and EUB338-Cy3 as negative control for yeasts; and (iv) test assays with the genus-specific probe Art1420-Cy3. In each assay 1000 cells were analysed in triplicate. Values represented correspond to the average of FC measurements and error bars to standard deviation (±SD).

#### 2.3.3. Experimental Evaluation of the Genus-Specific Probe for *Arthrobacter* spp.

Two sets of assays were performed to evaluate the performance and specificity of the Art1420 Cy3 probe.

(i) Stringency optimisation. Formamide concentration curves were constructed by performing RNA-FISH assays with Art1420 Cy3 using *Arthrobacter* sp. CCLBH BP301 (target) and *Bacillus* sp. CCLBH BP102 (non-target), both isolated from the same CH ecosystem. Formamide concentrations ([FA]) ranged from 0 to 35% (*v*/*v*). The percentage of fluorescent cells and fluorescence intensity (FI) were quantified by flow cytometry (FC), analysing 1000 cells per assay. Formamide curves were generated to determine the minimal [FA] that ensured high HE and FI for *Arthrobacter* cells, while maintaining minimal or undetectable fluorescence in *Bacillus* sp. The optimal hybridisation condition was defined as 10% (*v*/*v*) formamide.
(ii) Cross-reactivity assays. Using the optimised condition of 10% (*v*/*v*) formamide, Art1420 Cy3 was tested against CH-associated microorganisms: Arthrobacter sp. CCLBH BP301 and CCLBH BP302 (targets), and *Bacillus* sp. CCLBH BP102, *Rubrobacter radiotolerans* DSM 5868, *Rhodotorula* sp. CCLBH YMP502 and *Cryptococcus adeliensis* CCLBH YMP503 (non-targets). For each species, the four assay types described in Section 2.3.2 (blank, positive control, negative control and Art1420 Cy3 test) were performed. Epifluorescence microscopy (EM) and FC were used to assess the presence or absence of fluorescent signals, the percentage of fluorescent cells and their FI.

EM was used qualitatively to confirm the absence of signal in blanks and negative controls and the expected strong labelling in positive controls. FC provided quantitative HE and FI values for each probe–strain combination. In each assay, 1000 cells were analysed in triplicate. Reported values correspond to the mean of FC measurements, and error bars represent the standard deviation (±SD).

#### 2.3.4. Epifluorescence Microscopy and Flow Cytometry Analysis

Fluorescence images were acquired using a BA410E Motic microscope (Kowloon, Hong Kong) equipped with a 100 W quartz halogen Köhler illumination system (Kowloon, Hong Kong) with intensity control, an EF-UPRIII epi-illumination module, and a Moticam PRO 282B digital camera (Kowloon, Hong Kong). The microscope was fitted with Cy3 (ex D540/40, dm 565DCLP, em D605/55), FITC (ex D480/30, dm 505DCLP, em D535/40) and Cy5 (ex HQ620/60, dm Q660LP, em HQ700/75) filter sets, and images were captured and processed using Motic Images Plus 2.0LM software. Flow cytometry (FC) measurements were performed with a Muse^®^ Cell Analyzer (Merck, Darmstadt, Germany) and analysed using MuseSoft 1.4.0.0. software for FC analysis. Epifluorescence microscopy (EM) and FC analyses were carried out as described by Branco et al. (2020) [45]. EM was used to qualitatively assess the presence or absence of fluorescent signals within the microbial cells. In contrast, FC provided quantitative data, allowing the determination of both the percentage of fluorescent cells and their corresponding Fluorescence Intensities (FI).

For each assay type (blank, negative control, positive control, and test), FI was calculated as the sum of the fluorescence intensities of all fluorescent events within the gate. The percentage of fluorescent cells was calculated by correcting the counts obtained in each assay with the corresponding negative control.

For the positive control, the percentage of fluorescent cells (% FC*_pc_*) was determined as $(FC_{pc}-FC_{neg})/N\times 100$, where $FC_{pc}$ is the number of fluorescent events in the positive control, $FC_{neg}$ is the number of fluorescent events in the negative control (NONEUB338 or EUB338), and N is the total number of analysed cells (1000). For the negative control, the percentage of fluorescent cells (%FC*_neg_*) was calculated as $(FC_{neg}-FC_{blank})/N\times 100$, where $FC_{neg}$ is the number of fluorescent events in the negative control, $FC_{blank}$ is the number of fluorescent events in the blank. Similarly, for the test with the genus-specific probe, the percentage of fluorescent cells (%FC*_test_*) was calculated as $(FC_{test}-FC_{neg})/N\times 100$, where $FC_{test}$ corresponds to the number of fluorescent events in the Art1420-Cy3 assay and the other terms are as defined above.

### 2.4. Screening of Antimicrobial Activity of the 2–10 kDa Peptide Fraction from S. cerevisiae ISA 1028 Supernatant

#### 2.4.1. Production of the 2–10 kDa Peptide Fraction

Antimicrobial peptides (AMPs) produced by *S. cerevisiae* strains, previously reported by Albergaria et al. (2010) [59] and Branco et al. (2014, 2017, 2023) [49,50,51], were obtained from the supernatants of *S. cerevisiae* ISA 1028 cultures grown in synthetic juice prepared according to Branco et al. (2014) [49]. Briefly, *S. cerevisiae* ISA 1028 cells were grown at 25 °C, without agitation in synthetic juice at pH 4.5 (glucose 110 g/L, fructose 110 g/L; acids solution: tartaric acid 6 g/L, malic acid 3 g/L, citric acid 0.5 g/L; amino acids solution: yeast nitrogen base without amino acid 1.7 g/L, casamino acids 2 g/L, calcium chloride 0.2 g/L, arginine 0.8 g/L, proline 1 g/L, tryptophan 0.1 g/L, and 2.5 g/L yeast extract). All solutions were autoclaved, except the amino acid solution, which was sterilised by filtration (0.22 µm).

After seven days, cell-free supernatants were collected by filtration through 0.22 μm Millipore membranes (Merck Millipore, Algés, Portugal) and subsequently subjected to ultrafiltration using Amicon Ultra-15 centrifugal filter units (Merck Millipore, Algés, Portugal). The filtrates were first passed through 10 kDa molecular weight cut-off membranes to remove high-molecular-weight compounds and then concentrated 100-fold using 2 kDa membranes. The resulting 2–10 kDa peptide fraction was collected and screened for antimicrobial activity.

#### 2.4.2. Antimicrobial Tests

The antimicrobial activity of the 2–10 kDa peptide fraction obtained from *S. cerevisiae* ISA 1028 supernatants was evaluated against *Arthrobacter* sp. CCLBH-BP301 and *Arthrobacter* sp. CCLBH-BP302 at a total protein concentration of 1.5 mg/mL. A spectrophotometer (NanoDrop 2000, Thermo Fisher Scientific Inc., Waltham, MA, USA) was used to measure sample absorbance at 280 nm.

The antimicrobial assays were carried out in sterile 96-well microplates. Nutrient broth (Sigma-Aldrich, St. Louis, MO, USA, EUA) was used for *Arthrobacter* strains. Control assays for each strain consisted of the respective growth medium without the addition of the 2–10 kDa fraction.

The initial cell density was adjusted to 10^6^ cells/mL. Microplates were incubated at 30 °C with continuous shaking.

Cell growth was monitored by measuring absorbance at 600 nm using a Multiskan Go spectrophotometer (Thermo Fisher Scientific Inc., Waltham, MA, USA) and by enumerating colony-forming units (CFU) through classical plating throughout the 72 h incubation period. For CFU counts, 10 µL of samples were taken and after appropriate dilution (decimal serial dilution method) 100 µL were plated onto appropriate medium for each species. All bacteria described above were plated onto Nutrient Agar plates (Sigma-Aldrich, St. Louis, MO, USA, EUA). Whenever colonies could not be detected in agar plates inoculated with diluted samples, 100 µL of sample was directly plated onto agar plates. Thus, the detection limit of the CFU method for results presented in Section 3.3 was 10 CFU/mL. All assays were performed in triplicate.

**Figure 1:**
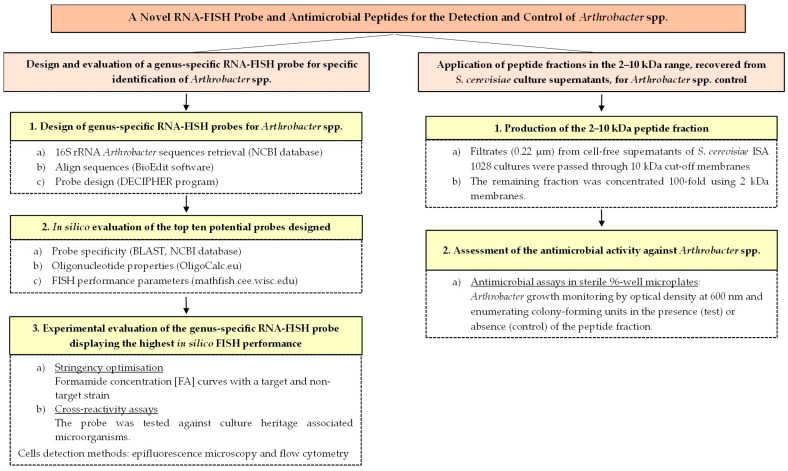
Conceptual workflow of the integrated strategy proposed for the detection and control of *Arthrobacter* spp. The diagram summarises the main stages of the study: (i) *in silico* design and evaluation of the genus-specific Art1420 RNA-FISH probe; (ii) experimental validation of probe performance by epifluorescence microscopy and flow cytometry; and (iii) assessment of the antimicrobial activity of the 2–10 kDa peptide fraction produced by *S. cerevisiae* ISA 1028 as a potential biocontrol tool against *Arthrobacter* spp.

To facilitate the visualisation of the overall study design and the relationship between the two complementary approaches explored, a schematic workflow is presented in Figure 1.

## 3. Results

### 3.1. In Silico Design and Evaluation of Genus-Specific Probe Candidates for Arthrobacter spp.

A genus-specific RNA-FISH probe for *Arthrobacter* spp. was designed using the DECIPHER Design Probes web tool. From 643 potential probe candidates generated for *Arthrobacter* spp., the ten highest-ranked sequences, based on specificity score and predicted hybridisation performance, were selected for *in silico* evaluation.

Three main analyses were conducted (Table 1): (a) probe specificity (matches with target organism) was reconfirmed through BLASTN searches on the NCBI database (https://blast.ncbi.nlm.nih.gov; [54]); (b) self-dimer, GC content, inter-molecular self-complementarity, and potential hairpin formation were assessed using OligoCalc [56]; and (c) *in silico* FISH simulations were carried out using mathFISH [57] to estimate hybridisation efficiency.

All ten top-ranked *Arthrobacter* spp. targeted probes displayed GC contents between 40 and 60%, a desirable range to minimise non-specific hybridisation, and short sequence lengths to enhance cell permeability (Table 1). None of the probes exhibited potential for self-dimer or hairpin formation.

Among these candidates, two probes, i.e., Art66 and Art1420, showed perfect matches to *Arthrobacter* spp. sequences without cross-reactivity with non-target microorganisms commonly found in cultural heritage (CH) environments. *In silico* predictions indicated that Art1420 had the highest theoretical hybridisation efficiency (100% at 0% formamide). Consequently, Art1420 was selected as the most promising genus-specific candidate for the specific and high-affinity detection of *Arthrobacter* cells.

### 3.2. Experimental Validation of the Art1420-Cy3 Probe

The performance and specificity of the Art1420-Cy3 probe were experimentally evaluated and optimised as described in Section 2.3.3.

Formamide concentration ([FA]) curves were constructed by performing RNA-FISH assays with the Art1420-Cy3 probe using a target (*Arthrobacter* sp. CCLBH-BP301) and a non-target microorganism (*Bacillus* sp. CCLBH-BP102) from the same ecosystem, with results analysed by flow cytometry. Hybridisation efficiency (HE) and fluorescence intensity (FI) were determined to identify the optimal [FA]% defined as the minimal formamide concentration (*v*/*v*) that combines high HE and strong FI for *Arthrobacter* spp. with minimal non-specific labelling of non-targets, while limiting exposure to formamide, a toxic and potentially hazardous reagent commonly used in FISH buffers [60].

**Figure 2:**
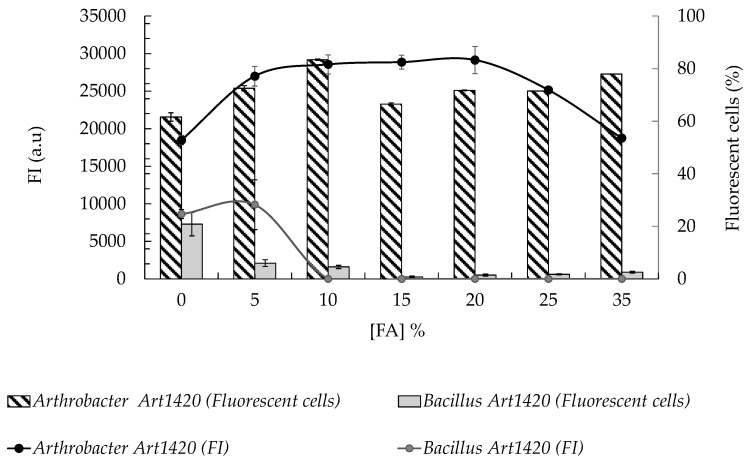
Flow cytometry results of RNA-FISH assays using genus-specific Art1420-Cy3 probe after treatment with increasing [FA]% (0–35%). Bars: percentage of fluorescent cells (%); lines: mean fluorescence intensity (FI, a.u.) for target (*Arthrobacter* sp. CCLBH-BP301, black) and non-target (*Bacillus* sp. CCLBH-BP102, grey) strains. For each condition, 1000 cells were analysed in triplicate; values correspond to the mean and error bars indicate standard deviation (±SD). Percentages of fluorescent cells were calculated considering NONEUB338 negative control results as base.

The formamide concentration curves revealed that the optimal hybridisation conditions for Art1420-Cy3 were achieved at [FA] = 10%. Since this [FA]% yields the highest percentage of fluorescent target cells (83.4%) with high FI (28562 a.u) for *Arthrobacter* sp. and no detectable fluorescence for *Bacillus* sp. cells (Figure 2).

**Figure 3:**
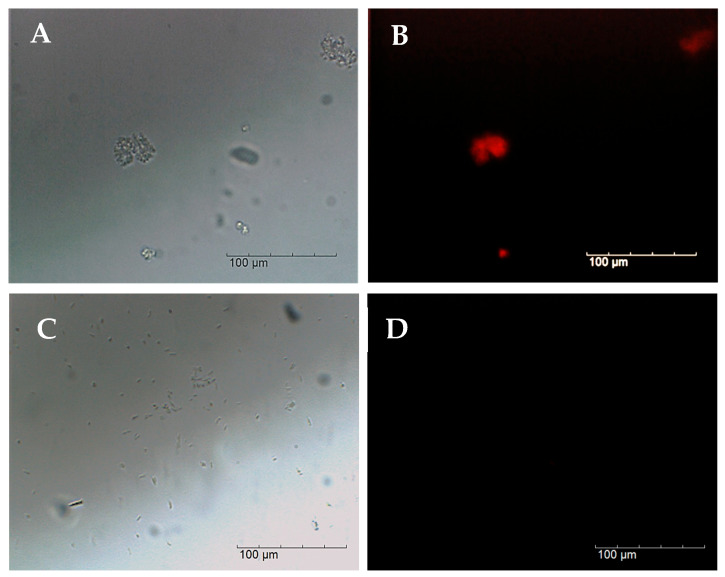
Photographs of *Arthrobacter* sp. CCLBH-BP 301 cells (**A**,**B**) and *Bacillus* sp. CCLBH-BP 102 after RNA-FISH treatment using Art1420-Cy3 probe (test assay) with 10% [FA] observed with the bright-field channel (**A**,**C**) and the Cy3 fluorescence channel (**B**,**D**).

The assays performed with the optimal [FA]% were also analysed by EM. Epifluorescence microscopy analysis of the RNA-FISH assays performed with 10% FA confirmed the specificity of the Art1420-Cy3 probe. The target cells (*Arthrobacter* sp. CCLBH-BP301) showed intense fluorescent signals (Figure 3B), whereas no probe-conferred fluorescence was observable for the non-target cells (*Bacillus* sp. CCLBH-BP102) (Figure 3D).

Once the hybridisation conditions that enabled specific detection of *Arthrobacter* cells using the Art1420-Cy3 probe were established, probe specificity was further evaluated through cross-reactivity tests against non-target organisms commonly found as heritage colonisers, i.e. *Bacillus* sp., *Rubrobacter radiotolerans*, *Rhodotorula* sp., and *Cryptococcus adeliensis*, along with additional tests using a second *Arthrobacter* sp. strain. FISH assays were performed following the in-suspension RNA-FISH protocol previously optimised by González-Pérez et al. (2017) [37].

**Figure 4:**
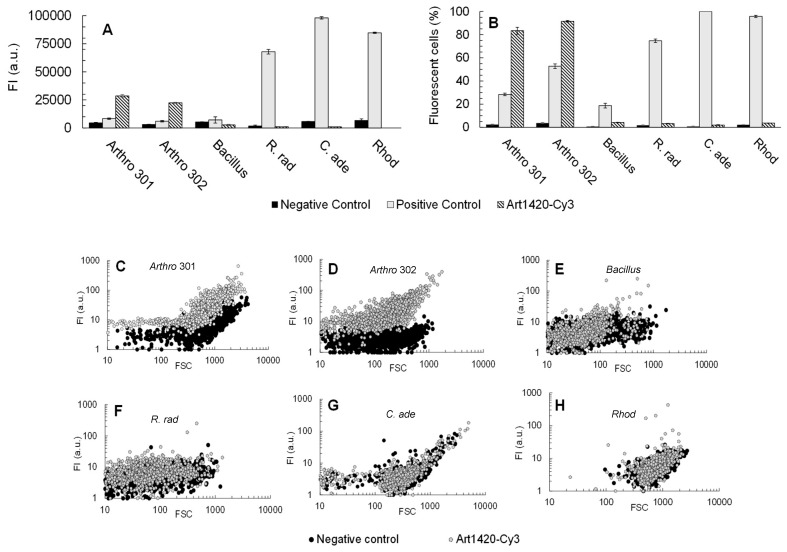
Flow cytometry results of fluorescent mean intensities (FI) (**A**) and percentage of fluorescent cells (**B**) after RNA-FISH treatment with [FA] = 10% for the species tested: *Arthrobacter* sp. CCLBH-BP 301 (Arthro 301), *Arthrobacter* sp. CCLBH-BP302 (Arthro 302), *Bacillus* sp. CCLBH-BP102 (*Bacillus*), *Rubrobacter radiotolerans* DSM 5868 (R. rad), *Cryptococcus adeliensis* CCLBH-YMP503 (C. ade), *Rhodotorula* sp. CCLBH-YMP502 (Rhod). The cells were permeabilized with EtOH 99% (*v*/*v*) and hybridised with NONEUB338-Cy3 (negative control), EUB338-Cy3 (positive control) and Art1420-Cy3. In each assay 1000 cells were analysed in triplicate. Values represented correspond to the average of FC measurements and error bars to standard deviation (±SD). Dot-plots probe-conferred fluorescence intensity/forward scattering (FI/FSC) directly given by the flow cytometry programme corresponding to *Arthrobacter* sp. CCLBH-BP301 (**C**), *Arthrobacter* sp. CCLBH-BP 302 (**D**), *Bacillus* sp. CCLBH-BP102 (**E**), *R. radiotolerans* DSM 5868 (**F**), *C. adeliensis* CCLBH-YMP503 (**G**) and *Rhodotorula* sp. CCLBH-YMP502 (**H**) hybridised with Art1420-Cy3 (grey dots) and NONEUB338-Cy3 (negative control, black dots).

Flow cytometry analysis (Figure 4A,B) showed that the Art1420-Cy3 probe, at a FA concentration of 10% (*v*/*v*), successfully labelled 83.4% and 91.6% of *Arthrobacter* spp. CCLBH-BP 301 and CCLBH-BP 302 cells, respectively, with high mean fluorescence intensities (28,562 a.u. and 22,413 a.u, respectively). In contrast, the assays with all non-target microorganisms exhibited less than 5% fluorescent cells, with low mean fluorescence intensities (below 500 a.u.). These findings confirm that Art1420-Cy3 displays high specificity and hybridisation efficiency, with no detectable cross-reactivity under the optimised hybridisation conditions (10% [FA]).

Figure 4C–H illustrates the dot plots of probe conferred fluorescence intensity versus forward scatter for each microorganism, highlighting the distribution of single cell fluorescence signals after hybridisation with Art1420-Cy3 probe (test) and the NONEUB338 probe (negative control). For *Arthrobacter* sp. CCLBH-BP301 and CCLBH-BP302 (Figure 4C,D) cells hybridised with the Art1420-Cy3 probe formed a well-defined population with high fluorescence intensities, clearly separated from the low-signal cluster corresponding to NONEUB338-hybridised cells. This pattern confirms that the majority of *Arthrobacter* cells were strongly and specifically labelled under the optimised hybridisation conditions. In contrast, *Bacillus* sp. CCLBH-BP102, *R. radiotolerans* DSM 5868. *C. adeliensis* CCLBH-YMP503, and *Rhodotorula* sp. CCLBH-YMP502 (Figure 4E–H) exhibited fluorescence distributions for cells hybridised with Art1420-Cy3 that largely overlapped with those obtained using the NONEUB338 probe, with only a few events showing slightly elevated signals. These results indicate the absence of significant cross-hybridisation and further confirm the high specificity of the Art1420-Cy3 probe toward *Arthrobacter* spp.

The RNA-FISH assays performed at 10% FA confirmed the high specificity of Art1420-Cy3: positive controls (EUB338-Cy3) showed expected labelling, negative controls (NONEUB338-Cy3) and blanks displayed absence of fluorescence, while the genus-specific Art1420-Cy3 probe generated intense signals in both *Arthrobacter* spp. strains and no detectable labelling in non-target bacteria and yeasts tested (Table 2). These results demonstrate that microscopy-based visual assessment under these conditions is sufficient to reliably discriminate *Arthrobacter* spp. from other common heritage colonisers.

Taken together, both epifluorescence microscopy and flow cytometry results demonstrate the potential of Art1420-Cy3 as a genus-specific FISH probe for *Arthrobacter* spp. detection under optimised conditions (10% [FA]).

These results highlight the promising potential of Art1420-Cy3 for specific *Arthrobacter* detection in CH conservation diagnostics. However, further validation is required for validating its real potential for the application in CH samples, which can include tests with cells in different growth phases and with mixed microbial communities as well as application in real heritage samples/biofilms.

### 3.3. Evaluation of the Antimicrobial Activity of the 2–10 kDa Peptide Fraction from S. cerevisiae ISA 1028 Supernatant

The 2–10 kDa peptide fraction obtained from *S. cerevisiae* ISA 1028 supernatants was evaluated for its antimicrobial activity against *Arthrobacter* sp. CCLBH-BP301 and CCLBH-BP302 strains.

**Figure 5:**
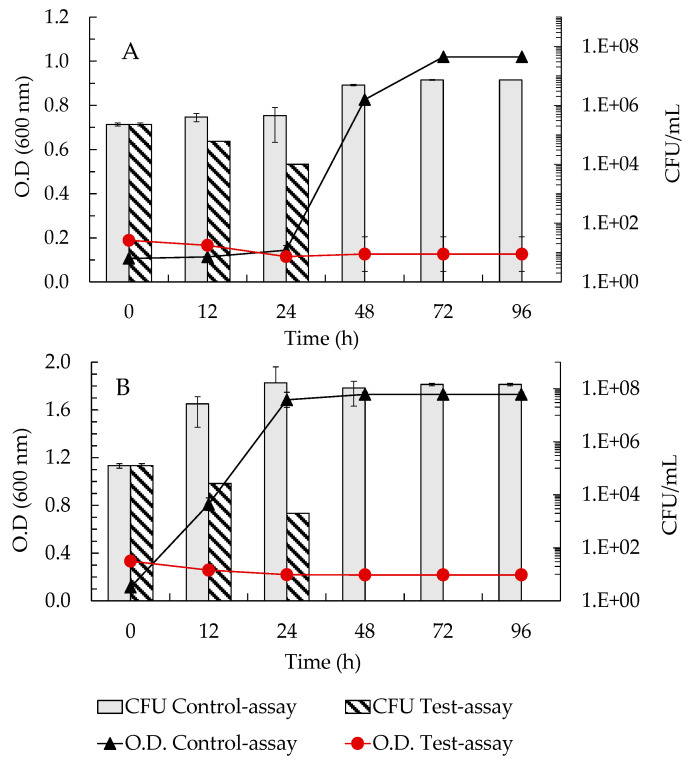
Culturability (CFU/mL) of (**A**)—*Arthrobacter* sp. CCLBH-BP 301; (**B**)—*Arthrobacter* sp. CCLBH-BP 302 in the absence (control-assay) and in the presence of the 2–10 kDa peptide fraction (Test-assay). Values represented correspond to the average of measurements and error bars to standard deviation (±SD).

Results showed that at a concentration of 1.5 mg/mL, the 2–10 kDa peptide fraction from *S. cerevisiae* ISA 1028 inhibited the growth of both *Arthrobacter* strains as shown by the marked reduction in optical density and culturability relative to untreated controls over the incubation period (Figure 5A,B). A pronounced bactericidal effect was observed, with culturability decreasing from approximately 10^8^ CFU/mL to the detection limit, approximately 10 CFU/mL, within 48 h in the presence of the peptide fraction, whereas control assays maintained high CFU counts throughout the experiment (Figure 5A,B).

## 4. Discussion

Several studies have combined FISH with flow cytometry to achieve rapid and accurate detection of specific bacterial groups in complex matrices, including environmental samples and probiotic products, consistently reporting high hybridisation rates and strong signal-to-background ratios when probe design and fixation/permeabilisation conditions are carefully optimised [61,62,63].

In particular, these studies have shown that Flow-FISH enables high-throughput analysis of target taxa while preserving single-cell resolution and allowing subsequent molecular characterisation of sorted populations. However, previous authors have also reported several methodological limitations, including reduced probe penetration in some bacterial taxa, variability in hybridisation efficiency depending on fixation and permeabilisation protocols, and background fluorescence arising from non-specific probe binding or autofluorescence in environmental matrices [44,64,65]. In the present study, the high proportion of fluorescent *Arthrobacter* spp. cells and the clear separation between target and non-target populations observed demonstrate that Art1420-Cy3 probe enables reliable, probe-based quantification of *Arthrobacter*. This approach shows potential to overcome the limitations of morphology- or pigment-based diagnostics, which frequently lead to misidentification of carotenoid-producing bacteria as pigmented yeasts such as *Rhodotorula* spp. [16]. Nevertheless, further validation in complex cultural heritage matrices and mixed microbial communities will be necessary to fully assess the applicability of this approach in real conservation scenarios.

While the Art1420-Cy3 RNA-FISH probe demonstrated high specificity and sensitivity for *Arthrobacter* spp. detection under laboratory conditions (83–92% hybridisation efficiency at 10% formamide), its performance relative to existing molecular tools merits consideration. Culture-based methods remain widely used in cultural heritage diagnostics but frequently underestimate microbial diversity due to the presence of viable but non-culturable (VBNC) cells and the selective growth of fast-growing microorganisms. PCR-based approaches, including 16S rRNA gene sequencing, provide high taxonomic resolution but require DNA extraction and typically detect both viable and non-viable cells. In contrast, the RNA-FISH approach enables direct *in situ* visualisation of individual microbial cells within their spatial context. Because FISH targets ribosomal RNA, which is generally more abundant in metabolically active cells, it can provide an indication of potentially active microbial populations while also enabling the detection of cells embedded within biofilms. Thus, the robust performance of the designed probe supports its application not only for the detection and quantification of *Arthrobacter* spp. by Flow-FISH, but also for their detection using other RNA-FISH approaches, potentially enabling *in situ* detection.

The concordance observed between epifluorescence microscopy (qualitative signal patterns) and flow cytometry (quantitative measurements of fluorescent cell percentages and fluorescence intensity) analysis further supports the robustness of both the probe and the hybridisation protocol for genus specific detection of *Arthrobacter* spp. [61,63]. Comparable agreement between microscopy and Flow-FISH analyses has been documented in studies involving the sorting and enrichment of functional guilds, such as methanotrophic bacteria and ultramicrobacteria, where high hybridisation efficiency and strong fluorescence signals are essential for reliable downstream analyses, including gene sequencing and functional assays [62,66,67]. Collectively, previous studies demonstrate that FISH-based flow cytometry can be integrated into advanced workflows for the isolation, enrichment, and molecular characterisation of specific microbial groups. In line with these findings, the Art1420-Cy3 probe shows promising potential to be incorporated into future pipelines for *Arthrobacter* spp. isolation, detection, or activity profiling within heritage environments. The performance of Art1420-Cy3 indicates its potential for the rapid and reliable identification of *Arthrobacter* spp. under controlled conditions. Its ability to discriminate *Arthrobacter* spp. from visually similar pigmented yeasts, such as *Rhodotorula* spp., highlights its prospective value for conservation diagnostics, particularly where chromatic similarities may complicate microbial identification [68,69,70]. However, additional studies involving mixed microbial communities, determination of detection limits, assessment of probe performance across different growth stages, and application to real heritage samples will be essential to fully validate its applicability [71,72,73].

Antimicrobial peptides produced by *S. cerevisiae* have attracted increasing attention as naturally derived antimicrobial compounds. Previous studies have shown that these peptides originate from metabolic processes, particularly from fragments derived from the glycolytic enzyme glyceraldehyde-3-phosphate dehydrogenase (GAPDH), can exhibit inhibitory activity against a broad range of bacterial and yeast species [49,50,51,52]. Peptide fractions in the 2–10 kDa range recovered from *S. cerevisiae* strains culture supernatants have previously demonstrated antimicrobial activity against wine-related bacteria such as *Oenococcus oeni* and *Lactobacillus* spp., as well as several pathogenic species, including *Escherichia coli, Listeria monocytogenes, Staphylococcus aureus* and *Salmonella* spp. [49,50,51,52].

The present findings extend this antimicrobial spectrum to include cultural heritage biodeteriogenic bacteria, demonstrating that the peptide fraction from *S. cerevisiae* displays broad-spectrum activity. Notably, this fraction exerted a rapid bactericidal effect on *Arthrobacter* spp., leading to a loss of cell viability within 48 h from 10^8^ to 10^1^ CFU/mL (Figure 5). From a conservation standpoint, these findings are promising, as they suggest that the peptide fraction could enable the rapid suppression of key bacterial biodeteriogens such as *Arthrobacter* spp., which are directly responsible for carotenoid-induced chromatic alterations on stone and mural paintings.

However, the antimicrobial assays performed in this study were conducted under laboratory conditions, and the behaviour of the peptide fraction on real cultural heritage substrates remains to be investigated. Further studies will therefore be required to evaluate application methods compatible with fragile materials, as well as the long-term stability and safety of these peptides in conservation contexts. Moreover, although the peptide fraction effectively suppresses viable *Arthrobacter* cells, neither this approach nor conventional chemical biocides removes carotenoid pigments that may already be deposited within porous materials such as limestone or mural plaster. Consequently, microbial suppression should be considered primarily a preventive intervention aimed at halting further microbial growth and pigment production, rather than a direct solution for removing existing chromatic alterations. The remediation of established stains generally requires complementary conservation treatments, such as controlled mechanical cleaning, laser cleaning, or other restoration techniques compatible with the affected substrate. Thus, the preliminary results of this study point to the potential applicability of the peptide fraction as a natural antimicrobial alternative to conventional chemical biocides [47,48,74].

Furthermore, the combination of this approach with the newly developed Art1420-Cy3 RNA-FISH probe for *Arthrobacter* detection may provide a novel dual strategy for heritage conservation. Because, together, these preliminary results point to a potential detection-and-control framework in which RNA-FISH using the Art1420-Cy3 probe could guide targeted diagnosis, while the 2–10 kDa peptide fraction could provide a microbial suppression. Further research is, however, necessary to confirm their applicability, safety, and long-term performance on heritage materials.

## 5. Conclusions

This study successfully developed a novel genus-specific RNA-FISH probe, Art1420-Cy3, demonstrating its high specificity and sensitivity for RNA-FISH detection of *Arthrobacter* spp. under optimised hybridisation conditions (10% FA). Additionally, the 2–10 kDa peptide fraction produced by *S. cerevisiae* ISA 1028 exhibited pronounced bactericidal activity against *Arthrobacter* spp., reducing culturability by over 4 log CFU/mL within 48 h.

The two complementary approaches explored the RNA-FISH probe Art1420-Cy3 and the *S. cerevisiae* peptide fraction, provides a promising foundation for an integrated framework combining specific detection and targeted control of *Arthrobacter* spp. in cultural heritage environments.

This study represents a significant step forward for overcoming longstanding diagnostic challenges associated with pigment-based misidentification of carotenoid-producing microorganisms on heritage surfaces. If further validated through testing on real heritage samples and mixed microbial communities, the combined applicability of both approaches could offer a novel strategy enabling precise, genus-level detection and monitoring of biodeteriogenic bacteria *Arthrobacter* sp. and sustainable bioremediation of cultural heritage when needed.

## Figures and Tables

**Table 1:** *In silico* analyses of the top ten scoring probes targeting *Arthrobacter* spp. generated by the DECIPHER design probes tool. The table lists each probe name and sequence together with the parameters used for its *in silico* evaluation: predicted probe efficiency, GC content, probe length, predicted hybridisation efficiency, existence of potential sites for hairpin formation or self-dimerisation, and BLASTN 2.16 matches with target and non-target organisms.

Name	Sequence (5′-3′)	Score ^a^	Efficiency ^b^	% GC Content	Length	% Hybridisation Efficiency ^c^	Potential Sites of:		Matches with	
							Hairpin	Self-Dimer	Target Organism (*Arthrobacter* spp.) ^d^	Non-Target Organisms Usually Found in CH Assets ^d^
Art1420	GGCTTCGGGTGTTACCA	0	0.677	58.80	17	100	none	none	330	0
Art66	CCCACAAGTGAGGTTCATCG	0	0.726	55.00	20	99.94	none	none	362	0
Art416	CTTCGGGTGTTACCAACTT	0	0.557	47.40	19	99.75	none	none	132	66
Art1060	GCATGATGATTTGACGTCGTC	0	0.616	47.60	21	98.53	none	none	22	1
Art726	CCAGAGACCTGCCTTC	0	0.677	60.00	15	97.75	none	none	18	2
Art283	CGGGGCACTTAATGCGT	0	0.703	58.80	17	92.80	none	none	10	9
Art769	GCACTTAATGCGTTAGCTACG	0	0.622	47.60	21	92.80	none	none	34	9
Art835	GCGGAAAACGTGGAATGTCCC	0	0.715	57.10	21	91.68	none	none	347	34
Art805	GCCCAACGTTTACGGCATG	0	0.731	52.60	19	81.09	none	none	145	58
Art1347	GCGTTGCTGATCTGCGATTA	0	0.732	50.00	20	77.43	none	none	3	24

^a^ Probes score calculated by DECIPHER programme: measures of the specificity of the probes to the target group. A score of zero is the highest specificity, while a more negative score is less specific. The individual probe’s specificity scores were calculated by the formula: specificity score = 0.2 × (number of potential non-targets) + Σ(1.2Δ[FA]m). Where Δ[FA]m is defined as the difference in formamide melt point between the target and non-target, and Σ refers to summing over all potential non-targets with Δ[FA]m ≥ −20%. The efficiency is the predicted efficiency of each of the probe permutation(s), which is required to be at least 50% at the input hybridisation conditions [56]. ^b^ Predicted probe efficiency calculated by DECIPHER program, which is required to be at least 50% at the input hybridisation conditions. ^c^ Hybridisation efficiency with [FA] = 0% predicted by mathFISH [57]. ^d^ Number of sequences with 100% of match identity found in 500 sequences in BLAST [54].

**Table 2:** Results obtained for the epifluorescence microscopy analysis of the RNA-FISH assays performed without probe and with NONEUB338-Cy3, EUB338-Cy3 and Art1420-Cy3 probes with [FA] = 10%. Signal intensities were classified into four categories: −, no signal; +, medium; +/+ high.

Microorganisms	Assay (Probe Used)			
	Blank (None)	Negative Control (NONEUB338-Cy3)	Positive Control (EUB338-Cy3)	Test (Art1420-Cy3)
*Arthrobacter* sp. CCLBH-BP 301	−	−	+	+/+
*Arthrobacter* CCLBH-BP 302	−	−	+	+/+
*Bacillus* sp. CCLBH-BP102	−	−	+	−
*Rubrobacter radiotolerans* DSM 5868	−	−	+/+	−
*Cryptococcus adeliensis* CCLBH-YMP503	−	−	+/+	−
*Rhodotorula* sp. CCLBH-YMP502	−	−	+/+	−

## Data Availability

The original contributions presented in this study are included in the article. Further inquiries can be directed to the corresponding author.
